# Recent progress and perspectives on the mechanisms underlying Asbestos toxicity

**DOI:** 10.1186/s41021-021-00215-0

**Published:** 2021-10-12

**Authors:** Akio Kuroda

**Affiliations:** Unit of Biotechnology, Graduate School of Integrated Sciences for Life, Hiroshima University, 1-3-1 Kagamiyama, Higashi Hiroshima, Hiroshima, 739-8530 Japan

**Keywords:** Asbestos, Toxicity, Mesothelioma, Carcinogenesis, Mutagenic microenvironment, *BAP1*, Live-cell imaging

## Abstract

Most cases of mesothelioma are known to result from exposure to asbestos fibers in the environment or occupational ambient air. The following questions regarding asbestos toxicity remain partially unanswered: (i) why asbestos entering the alveoli during respiration exerts toxicity in the pleura; and (ii) how asbestos causes mesothelioma, even though human mesothelial cells are easily killed upon exposure to asbestos. As for the latter question, it is now thought that the frustrated phagocytosis of asbestos fibers by macrophages prolongs inflammatory responses and gives rise to a “mutagenic microenvironment” around mesothelial cells, resulting in their malignant transformation. Based on epidemiological and genetic studies, a carcinogenic model has been proposed in which BRCA1-associated protein 1 mutations are able to suppress cell death in mesothelial cells and increase genomic instability in the mutagenic microenvironment. This leads to additional mutations, such as *CDKN2A* [p16], *NF2, TP53, LATS2,* and *SETD2*, which are associated with mesothelioma carcinogenesis. Regarding the former question, the receptors involved in the intracellular uptake of asbestos and the mechanism of transfer of inhaled asbestos from the alveoli to the pleura are yet to be elucidated. Further studies using live-cell imaging techniques will be critical to fully understanding the mechanisms underlying asbestos toxicity.

**Supplementary Information:**

The online version contains supplementary material available at 10.1186/s41021-021-00215-0.

## Background

**Fig. 1:**
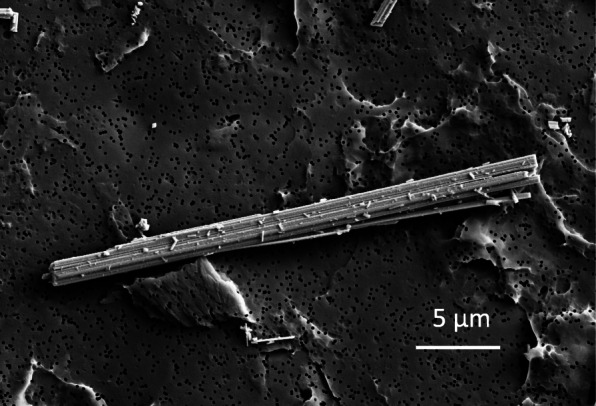
Electron micrograph of asbestos. Amosite, obtained from the Japan Association for Working Environment Measurement (Tokyo, Japan), was observed by field emission scanning electron microscopy (Ultra Plus, Carl Zeiss)

Asbestos is composed of bundles of fine silicate fibers (Fig. 1) and has been industrially used for its fire resistance, heat insulation, and strength. However, asbestos fibers are released into the air as asbestos-containing materials undergo mechanical damage or deterioration over time. The inhalation of asbestos fibers damages the lungs and can cause serious health problems, such as pleural mesothelioma and lung cancer [1–3]. The incidence of mesothelioma increased markedly after World War II when large amounts of asbestos were used [4]. The widespread use of asbestos continued until the early 1980s in the United States and Europe. By 1990, most developed countries reduced the use of asbestos. However, it continues to be used in several countries, including India, China, and Russia. The World Health Organization (WHO) estimates that approximately 125 million people worldwide are exposed to asbestos in the workplace [5]. In countries where this is the case, the incidence and mortality due to mesothelioma are expected to increase.

While asbestos has been banned in most developed countries, large quantities of asbestos-containing materials remain in old buildings, and people are at risk of exposure to asbestos during demolition. In addition, natural minerals, such as talc, a raw material used in the manufacture of cosmetics, pharmaceuticals, and baby powder, may also contain asbestos. It has recently been highlighted that asbestos-contaminated talc may cause cancer [6, 7]. According to the statistics reported by Global Burden of Diseases and Injury in 2016, the death toll due to mesothelioma in Japan and the United States remains high, at approximately 1500 and 3300, respectively [8]. Therefore, even years after the ban on the use of asbestos, the problem remains partly unresolved.

### Asbestos toxicity

Asbestos is composed of six types of natural mineral fibers: chrysolite, amosite, crocidolite, actinolite, tremolite, and anthophyllite. In nature, there are 400 other types of mineral fibers, some of which are carcinogenic and associated with mesothelioma. Erionite, for example, is known to be a carcinogenic fiber and is associated with the mesothelioma epidemic in the village of Cappadocia, Turkey [9–11]. In contrast, palygorskite, which is present in desert dust, is reported to be non-carcinogenic [12]. The toxicity of a mineral fiber is related not only to the chemical composition of the mineral but also to its surface reactivity, crystallinity, and the presence of transition metals [13]. The size and shape of the fibers are also important since they affect whether the inhaled fibers penetrate the alveolar space through the airways [13]. The stability of the fibers in the lungs can greatly affect their toxicity since ones that reach the alveolar space may be degraded or removed by macrophages for detoxification.

The “mechanical interference mechanism” model was previously proposed, which predicted that phagocytosed asbestos fibers mechanically interact with the mitotic spindle and cause chromosomal changes, leading to carcinogenesis [14]. However, it was reported that human mesothelial cells were inevitably killed within 2–10 days of asbestos exposure, and no immortalized cell lines that could evolve into cancers were detected [15, 16]. The mechanical interference mechanism is paradoxical since mesothelial cells are killed before they are transformed into cancer cells. The latency period of asbestos-induced mesothelioma is known to be 30–50 years. Therefore, a long-term, chronic inflammatory asbestos-induced mechanism is considered the leading cause of carcinogenesis [17–20].

### Frustrated phagocytosis and continuous inflammation

**Fig. 2:**
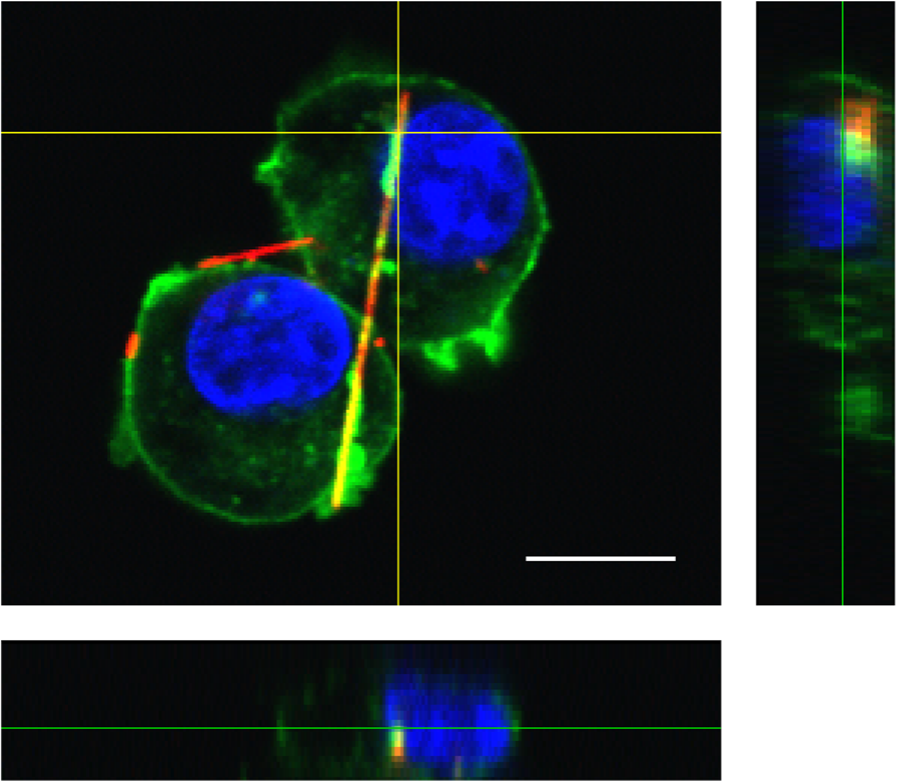
Frustrated phagocytosis. Fluorescently labeled asbestos (red) was phagocytosed by RAW264.7 cells. The actin cytoskeletons and nuclei were stained with Actin Green™ 488 (green) and 4′,6-diamidino-2-phenylindole (blue), respectively. This image was taken using a confocal laser scanning microscope [81]. Scale bar = 10 μm. This figure has been reproduced from the open-access article [81]

Alveolar macrophages phagocytose particles, such as dust and microorganisms, and remove them from the surface of the alveoli. Alveolar macrophages also attempt to remove asbestos fibers via phagocytosis [21, 22]. It is reported that relatively short fibers appear to be completely encapsulated and removed by the phagosomes, so fibers less than 5 μm in length are not retained in the lungs and do not cause chronic inflammation [23]. In contrast, longer fibers are imperfectly phagocytosed by macrophages, resulting in “frustrated phagocytosis” (Fig. 2), and can remain in the lungs for extended periods [24, 25]. Long asbestos fibers are known to be associated with carcinogenesis [26]. Phagocytosed asbestos fibers activate the NOD-like receptor family pyrin domain containing 3 (NLRP3) inflammasome and trigger the production of the inflammatory interleukin-1ß (IL-1ß) [27]. Therefore, long fibers retained in the lungs cause chronic inflammation, exerting a pleiotropic effect and resulting in malignant transformation. Since the toxicity of asbestos depends on the shape of the fibers, the WHO definition of asbestos is based on not only the mineralogy but also the fiber dimensions (length ≥ 5 μm, width ≤ 3 μm, and aspect ratio ≥ 3).

The NLRP3 inflammasome responds to diverse numbers and structures of exogenous stimuli, including crystalline and particulate matter, extracellular adenosine triphosphate, RNA-DNA hybrids, and various pathogens. Intraperitoneal injections of multi-walled carbon nanotubes (CNTs) have been reported to cause massive granulomatous inflammation in the diaphragms of wild-type mice [28]. Palomaki et al. found that both CNTs and asbestos induce the activation of the NLRP3 inflammasome in human macrophages [29]. Similarly, various crystals, such as silica, urate monohydrate crystals, hydroxyapatite, cholesterol, alum crystals, and nanomaterials such as TiO_2_ nanoparticles, have also been reported to induce NLRP3 inflammasome activation in macrophages [30–34].

The molecular mechanism underlying inflammasome activation has been extensively studied [35–38]. At least two signals are required for the activation of the NLRP3 inflammasome: the first signal (signal 1) induces the expression of NLRP3, along with pro-IL-1ß, via nuclear factor-ϰB; the second signal (signal 2) stimulates the assembly of multiple protein complexes, including NLRP3, apoptosis-associated speck-like protein containing a C-terminal caspase recruitment domain (ASC), and procaspase-1, resulting in the activation of caspase-1 protease. Subsequently, the active caspase-1 processes pro-IL-1ß into mature IL-1ß, which is released extracellularly through the damaged macrophage membrane. Upon recognition of the crystal, macrophage surface receptors transmit signals 1 and/or 2. It is also proposed that the receptor-independent recognition of crystals may transmit signal 2 [39].

### Asbestos receptors

Scavenger receptors, which are present only on macrophages and some endothelial cells, are involved in the recognition of a broad range of ligands. Class A scavenger receptors, such as class A scavenger receptor 1 and macrophage receptor with collagenous structure (MARCO), are known to bind to silica and titanium particles [40]. Murthy et al. reported that lower levels of fibrosis were seen in MARCO-deficient mice after exposure to chrysotile asbestos [41], indicating that MARCO may contribute, in part, to asbestos-induced pulmonary fibrosis. Recently, class B scavenger receptor member 1 (SR-B1) has been identified as a novel silica receptor [42]. SR-B1 could also act as an additional receptor for asbestos; however, this still needs to be confirmed experimentally. Meanwhile, an apparently unrelated receptor has been found to recognize particulate matter. Interestingly, T-cell membrane protein 4, a phosphatidylserine receptor, has been reported to bind to CNTs and facilitate their cellular uptake [43].

### Carcinogenesis

#### (i) Mutagenic microenvironment

**Fig. 3:**
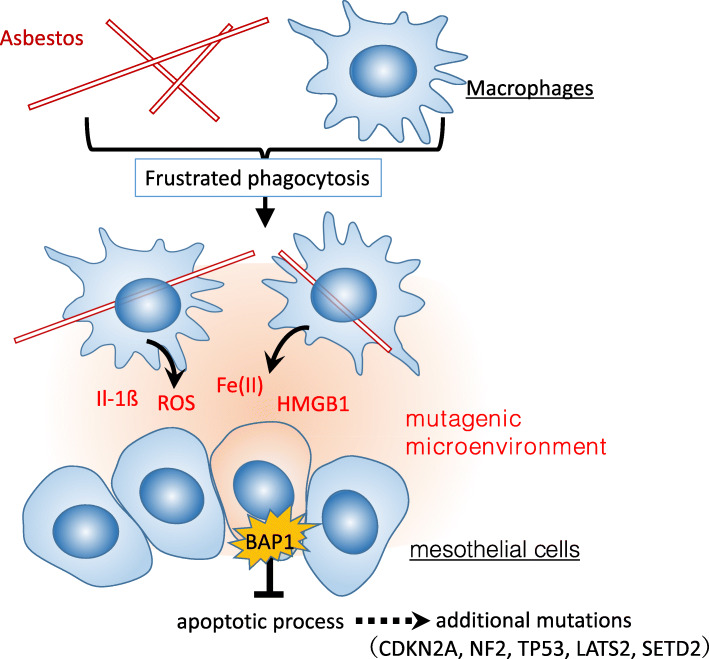
A model of the carcinogenesis of mesothelioma in an asbestos-induced mutagenic microenvironment. Phagocytosed asbestos induces a mutagenic microenvironment rich in Fe (II), IL-1ß, ROS, and HMGB1 [16, 17, 19, 27, 46, 50–52]. The *BAP1* mutation contributes to the suppression of mesothelial cell death and the accumulation of additional mutations associated with mesothelioma carcinogenesis [69]. Abbreviations: IL-1ß: Interleukin-1ß; ROS, reactive oxygen species; HMGB1, high mobility group box-1 protein; BAP1, BRCA1-associated protein 1

Long asbestos fibers that reach the pleura are retained for extended periods of time [24, 25] and cause prolonged inflammation due to frustrated phagocytosis. The activated NLRP3 inflammasome induces the secretion of IL-1ß [27]. Damaged and necrotic cells release inflammatory proteins, such as high mobility group box-1 protein (HMGB1), which induce the accumulation of macrophages and the activation of the NLRP3 inflammasome, resulting in amplification of the inflammatory response and secretion of tumor necrosis factor-⍺ [19]. Inflammatory cells release reactive oxygen species (ROS) and reactive nitrogen species (RNS), which are capable of causing DNA damage [44, 45] (Fig. 3). Blake et al. found that asbestos fiber internalization generates a significant increase in intracellular ROS [46]. Xu et al. demonstrated that ROS mediate asbestos-induced DNA damage mutagenesis in human-hamster hybrid cells [17].

The histological diagnosis of asbestosis toxicity requires the presence of “an asbestos body,” a core asbestos fiber coated with iron-containing materials. Therefore, asbestos toxicity is associated with iron [20, 47]. Intraperitoneal injection of ferric saccharate (glucaric acid-iron conjugate) can induce malignant mesothelioma [48, 49], suggesting that excess iron is involved in the carcinogenesis of mesothelioma. Toyokuni et al. proposed that macrophage necrosis occurs repeatedly along with lysosomal cell death [50], and ferroptosis [51] could establish an Fe (II)-rich mutagenic microenvironment [52], although they need to elucidate mutations induced under these conditions.

#### (ii) suppression of cell death by BRCA1-associated protein 1 (*BAP1*) mutations

It has been proven in three remote villages in Cappadocia, Turkey, that genetic predisposition is involved in the pathogenesis of mesothelioma. In 2001, Carbone et al. revealed that mesothelioma occurred primarily in only a subset of families in these villages, but not in others [11]. In 2011, the team conducted a study in two families in the United States who had not been exposed to asbestos; they found that the *BAP1* mutation was associated with a high incidence of mesothelioma [53]. Among the somatic mutations occurring during tumor cell proliferation, *BAP1* mutations have been observed in approximately 60% of mesotheliomas [54–58]. *BAP1* mutations are known to cause various types of cancers, including mesothelioma, and are referred to as “*BAP1* tumor predisposition syndrome” [59]. Genome analysis of mesothelioma has also revealed mutations in the *CDKN2A* [p16], *NF2, TP53, LATS2*, and *SETD2* genes [57, 60]. In a mouse model, two pathways controlled by *CDKN2* and *TP53* are believed to be critical to the development of mesothelioma [61].

The BAP1 protein is a deubiquitylase that modulates the activity of multiple genes and proteins that control DNA replication, DNA repair, metabolism, and cell death [62, 63]. Bononi et al. reported that BAP1 regulates both DNA repair and apoptosis following DNA damage caused by asbestos, ultraviolet light, radiation, or chemotherapy [64, 65]. Zhang et al. reported that cells with reduced BAP1 activity are less prone to ferroptosis [66], indicating that *BAP1* mutant cells can escape cell death [67]. Xue et al. noted that HMGB1 secreted during necrosis induces autophagy and suppresses apoptosis, leading to the accumulation of mutations associated with carcinogenesis [68].

It is known that no cancer cell lines arise from mesothelial cells in vitro upon exposure to asbestos [69]. Carbone et al. proposed a model that could explain the paradox of asbestos carcinogenicity [69]. First, the phagocytosed asbestos in macrophages may cause a mutagenic microenvironment rich in ROS and HMGB1, enhancing mutations in mesothelial cells. Then, *BAP1*-mutated mesothelial cells may escape cell death and accumulate further DNA damage (*CDKN2A, NF2, TP53, LATS2, SETD2*), leading to carcinogenesis [67, 69] (Fig. 3). This model explains how mesothelial cells that have a short lifespan may form mesotheliomas after long-term inflammatory disorders [69]. In addition, Otsuki et al. have investigated the mechanisms of asbestos toxicity from the aspect of antitumor immunity [70, 71]. They found that T lymphocytes-dependent antitumor immunity was attenuated by the gradual exposure of asbestos to immune cells. This slow attenuation over a period of 30 to 50 years may contribute to malignant transformation in mesothelial cells.

### Dynamic analysis of asbestos by live-cell imaging

**Fig. 4:**
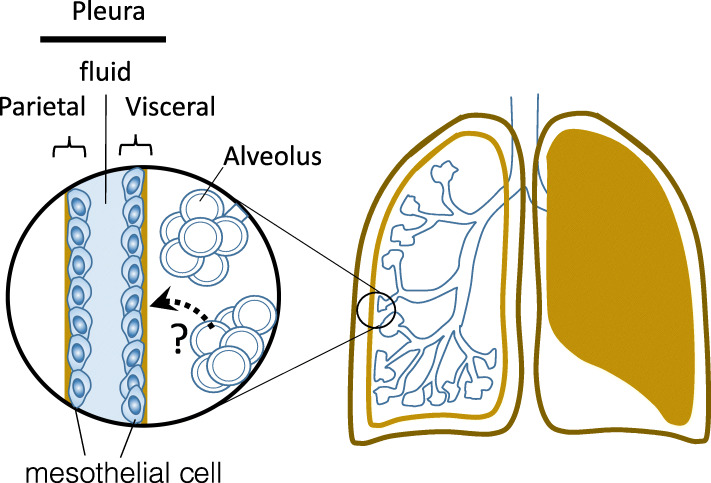
Transport of asbestos fibers from the alveoli to the pleura

It remains unclear why asbestos entering the alveoli during aspiration exerts toxicity in the pleura. Once asbestos fibers reach the pleura, they remain in the pleura due to their physical size. Therefore, the primary question that must be answered is how the asbestos fibers reach the pleura (Fig. 4) [31, 72–74]. Since alveolar macrophages have been implicated in the transfer of substances to the extracellular space or blood [75], macrophages may also be responsible for transferring asbestos fibers to the pleura.

The dynamic biological processes that occur during and after the internalization of asbestos fibers are not fully understood. This may partly be due to the relative lack of developments in dynamic analytical techniques for the analysis of asbestos toxicity. Several studies have analyzed the intracellular internalization of asbestos fibers by transmission electron microscopy or a combination of atomic force microscopy and soft X-ray microscopy [46, 76–78]. These methods provide more detailed information than light microscopy but are not suitable for analyzing dynamic biological processes, such as those occurring during the phagocytosis of asbestos and the co-localization of biomolecules. A popular strategy for conducting dynamic analysis is live-cell imaging, using fluorescence microscopy. My collaborators and I have developed asbestos-binding protein-based fluorescent probes for environmental monitoring [79, 80] and live-cell imaging [81]. The finest fibers of chrysotile asbestos visualized by fluorescence microscopy are approximately 30–35 nm in diameter (estimated by scanning electron microscopy), which is similar to the dimensions of a single chrysotile fibril [82]. Fluorescently labeled asbestos and live-cell imaging are used in combination with confocal laser scanning microscopy to dynamically analyze the phagocytosis of asbestos (Fig. 2) and subsequent biological processes [81]. These techniques demonstrated that macrophages carrying internalized asbestos were motile (Video 1), indicating the involvement of macrophages in the transfer of asbestos to the pleura. In the future, such techniques may be used to elucidate the asbestos receptors involved in the pathogenesis and the mechanisms involved in asbestos transfer in vivo.

## Conclusions

Paradoxical questions related to asbestos toxicity are explained by the frustrated phagocytosis of asbestos fibers by macrophages, which provide “a mutagenic microenvironment” around mesothelial cells and induce the *BAP1* mutation. This mutation can contribute to the suppression of mesothelial cell death and the accumulation of additional mutations involved in mesothelioma carcinogenesis. The intracellular uptake of asbestos and the mechanisms involved in the transfer of inhaled asbestos must be fully understood in order to determine the mechanism underlying asbestos toxicity.

## Supplementary Information

**Additional file 1: Video 1** Motility of macrophages that phagocytose asbestos (red). The detailed experimental conditions are described in reference [81].

## Data Availability

All data that were analyzed and reviewed are included in this article.

## Abbreviations

BAP1: BRCA1-associated protein 1
WHO: World Health Organization
NLRP: Nucleotide-binding oligomerization domain, leucine-rich repeat, and pyrin domain-containing
IL-1ß: Interleukin-1ß
CNT: carbon nanotube
ASC: apoptosis-associated speck-like protein containing a C-terminal caspase recruitment domain
SR-B1: Class B scavenger receptors 1
HMGB1: High mobility group box-1 protein
ROS: Reactive oxygen species
MARCO: macrophage receptor with collagenous structure
